# Eradication of Chronic Myeloid Leukemia Stem Cells: A Novel Mathematical Model Predicts No Therapeutic Benefit of Adding G-CSF to Imatinib

**DOI:** 10.1371/journal.pcbi.1000503

**Published:** 2009-09-11

**Authors:** Jasmine Foo, Mark W. Drummond, Bayard Clarkson, Tessa Holyoake, Franziska Michor

**Affiliations:** 1 Computational Biology Program, Memorial Sloan-Kettering Cancer Center, New York, New York, United States of America; 2 Section of Experimental Haematology, Medical Faculty, University of Glasgow, Glasgow, United Kingdom; 3 Molecular Pharmacology and Chemistry Program, Memorial Sloan-Kettering Cancer Center, New York, New York, United States of America; Imperial College London, United Kingdom

## Abstract

Imatinib mesylate induces complete cytogenetic responses in patients with chronic myeloid leukemia (CML), yet many patients have detectable BCR-ABL transcripts in peripheral blood even after prolonged therapy. Bone marrow studies have shown that this residual disease resides within the stem cell compartment. Quiescence of leukemic stem cells has been suggested as a mechanism conferring insensitivity to imatinib, and exposure to the Granulocyte-Colony Stimulating Factor (G-CSF), together with imatinib, has led to a significant reduction in leukemic stem cells *in vitro*. In this paper, we design a novel mathematical model of stem cell quiescence to investigate the treatment response to imatinib and G-CSF. We find that the addition of G-CSF to an imatinib treatment protocol leads to observable effects only if the majority of leukemic stem cells are quiescent; otherwise it does not modulate the leukemic cell burden. The latter scenario is in agreement with clinical findings in a pilot study administering imatinib continuously or intermittently, with or without G-CSF (GIMI trial). Furthermore, our model predicts that the addition of G-CSF leads to a higher risk of resistance since it increases the production of cycling leukemic stem cells. Although the pilot study did not include enough patients to draw any conclusion with statistical significance, there were more cases of progression in the experimental arms as compared to continuous imatinib. Our results suggest that the additional use of G-CSF may be detrimental to patients in the clinic.

## Author Summary

Imatinib mesylate (Gleevec) is currently the standard treatment for chronic myeloid leukemia (CML) and elicits a large reduction in leukemic cell burden in most patients. However, strong evidence suggests that imatinib does not cure the disease; approximately 20% of patients relapse within three years, and discontinuation of imatinib therapy often leads to a rebound of the leukemic cell burden. Laboratory studies have suggested that there exists a subpopulation of “quiescent” leukemia cells (i.e., cells that do not divide) that may be insensitive to imatinib treatment. It has been postulated that the disease outcome may be improved by administering imatinib in conjunction with the Granulocyte-Colony Stimulating Factor (G-CSF), a growth factor which “wakes up” the quiescent stem cells and sensitizes them to imatinib. In this study, we design a novel mathematical model of stem cell quiescence to investigate the treatment response to imatinib and G-CSF. We find that adding G-CSF to an imatinib treatment protocol leads to observable effects only if the majority of leukemic stem cells are quiescent. Our model also predicts that adding G-CSF leads to a higher risk of resistance, since it increases the number of leukemic stem cell divisions and thus the probability of acquiring a resistance mutation.

## Introduction

The existence of cancer stem cells, a rare subpopulation of cancer cells responsible for tumor initiation and maintenance, was first postulated in the 1960s [1]. In leukemia in particular, increasing evidence suggests that leukemic stem cells are the only cells within the tumor capable of propagating the disease [2]–[6]. Leukemic stem cells share many properties – such as self-renewal, pluripotency, and quiescence – with tissue stem cells, and they appear to remain untouched by both conventional chemotherapy and targeted drugs [6]. Since the repopulating capabilities of leukemic stem cells necessitate their eradication for a cure of the disease, the development of new therapeutic approaches targeting leukemic stem cells would have a profound impact on cancer management.

Chronic myeloid leukemia (CML) is associated with the Philadelphia chromosome, which results from a reciprocal translocation between chromosomes 9 and 22 generating the BCR-ABL fusion oncogene [7],[8]. The ABL kinase inhibitor imatinib greatly improves outcome in CML patients [9]; however, some evidence suggests that it cannot eradicate the disease since it preferentially targets progenitor cells while being incapable of depleting leukemic stem cells [10]. Surviving leukemic stem cells are a potential source of relapse, as demonstrated by the dynamics of leukemic cells in patients who discontinue imatinib after a prolonged administration of therapy [11]–[17]. Several mechanisms of leukemic stem cell insensitivity to ABL kinase inhibitors have been suggested [18]; those mechanisms include quiescence of leukemic stem cells, drug export from the cytoplasm, overexpression of BCR-ABL in stem cells as compared to differentiated cells, and a lack of immune responses against leukemic stem cells.

Under normal circumstances, a fraction of hematopoietic stem cells is quiescent [19]–[24]. Cycling and quiescent hematopoietic stem cells display major functional differences, mostly reflected in their homing and mobilization abilities [25]–[29]. Quiescent stem cells are mobilized by Granulocyte-Colony Stimulating Factor (G-CSF) and other agents and show preferential homing to the bone marrow as compared to dividing hematopoietic stem cells [29]. In contrast, CML stem cells are constitutively present in the circulation, but also contain a subpopulation of quiescent cells [30]. This quiescent subpopulation has been shown to be insensitive to imatinib therapy *in vitro* and might therefore represent a therapeutically relevant subpopulation of cancer cells [31]. However, the extent of quiescence of CML stem cells and the *in vivo* response of such cells to imatinib have not yet been established.

In this paper, we present a novel mathematical model to investigate the response of cycling and quiescent leukemic stem cells to treatment with imatinib alone or imatinib combined with G-CSF. We use clinical data of a Phase II trial administering imatinib and G-CSF and study the effects of various treatment strategies on the leukemic stem cell pool [32]. This study is part of a growing literature of theoretical approaches to CML [16], [33]–[39].

## Methods

### Mathematical Modeling of the CML Cell Hierarchy, Turnover, and Response to Imatinib

**Figure 1:**
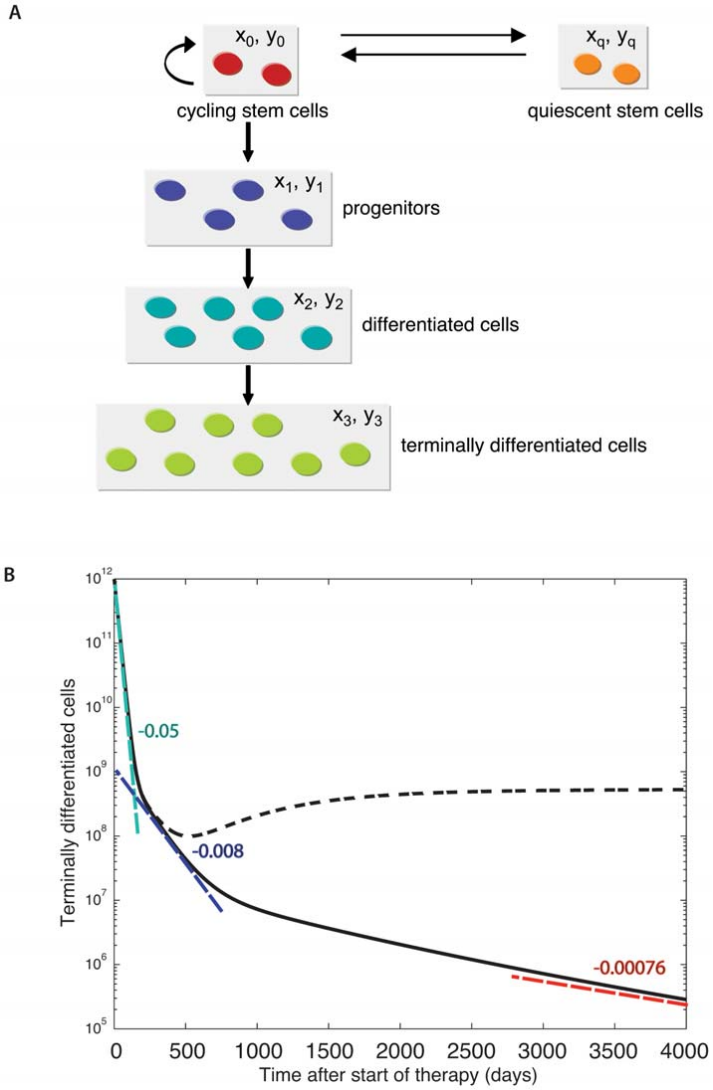
A mathematical model of chronic myeloid leukemia. (A) The model contains different subpopulations of the differentiation hierarchy of hematopoietic cells. On top of the hierarchy, there are cycling and quiescent stem cells. Cycling stem cells can reproduce and also produce progenitor cells. Progenitors give rise to differentiated cells, which produce terminally differentiated cells. (B) We show the simulation results of the terminally differentiated leukemic cell population, 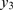, during imatinib treatment which initiates on day 0. The leukemic cell burden declines in a multi-phasic manner: terminally differentiated leukemic cells decrease at their death rate, 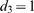 per day, until they reach a steady state with differentiated leukemic cells, then they track the latter's disease kinetics (slope not shown for reasons of low resolution of the data). Differentiated leukemic cells decline at their death rate, 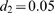 per day, until they reach an equilibrium with leukemic progenitors (green slope). Progenitors decline at their death rate, 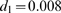 per day, until they reach an equilibrium with leukemic stem cells (blue slope). These slopes are estimated from the IRIS data [16]. Leukemic stem cells increase or decline at a rate equal to the asymptotic value of 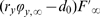. In this figure, we show examples of the kinetics in which leukemic stem cells are assumed to decrease during imatinib therapy (red slope), and in which leukemic stem cells continue to increase during therapy (dashed line). Parameter values are 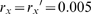, 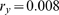, 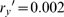, 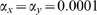, 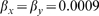, 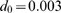, 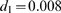, 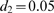, 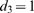, 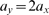, 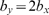, 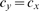, 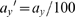, 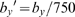, and 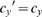. The other parameters are calculated such that in equilibrium, there are 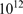 normal terminally differentiated cells, 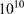 normal differentiated cells, 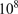 normal progenitors, and 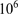 normal and 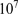 leukemic stem cells (cycling plus quiescent). All parameters whose values during therapy are not specified are unaltered by treatment. Our parameter choices set the initial equilibrium frequency of both normal and leukemic cycling stem cells to 90% (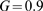).

Consider a differentiation hierarchy of hematopoietic cells. On top of the hierarchy, there are cycling and quiescent stem cells. Quiescent stem cells can become cycling stem cells, while the latter can become quiescent stem cells. Cycling stem cells give rise to progenitor cells, which produce differentiated cells, which in turn produce terminally differentiated cells (Fig. 1A). This differentiation hierarchy applies to normal and leukemic cells [40]. The abundances of normal cycling and quiescent stem cells, progenitors, differentiated, and terminally differentiated cells are denoted by 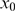, 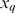, 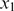, 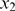, and 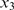; the respective abundances of leukemic cells are denoted by 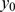, 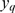, 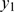, 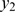, and 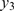. Normal and leukemic cycling stem cells divide at rates 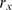 and 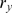, respectively. The rates at which cycling stem cells produce quiescent stem cells and vice versa are denoted by 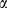 and 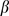. The rate constants for the production of progenitors, differentiated cells and terminally differentiated cells are given by *a*, *b*, and *c*. Cycling stem cells die at rate 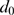, progenitors at rate 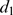, differentiated cells at rate 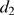, and terminally differentiated cells at rate 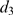 per day. Here we assume that cells at all levels may reproduce symmetrically and/or asymmetrically; the limited replication potential of more differentiated cell types can be considered as part of the differentiation rates. The BCR-ABL oncogene is present in all leukemic cells and leads to a slow clonal expansion of cycling leukemic stem cells; the latter effect is assumed since otherwise, leukemic stem cells could not make up a significant fraction of the stem cell compartment at diagnosis [30],[41]. Furthermore, BCR-ABL increases the rate at which leukemic progenitors are produced. The basic model is displayed in Table 1. Density dependence is achieved by the functions 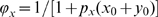 and 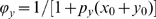; these functions ensure that normal and leukemic stem cells remain at a constant abundance once the system has reached a steady state. To achieve realistic equilibrium conditions, we set the constants 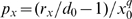 and 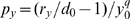, where 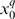 and 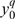 are the equilibrium abundances of normal and leukemic stem cells, respectively. In the absence of leukemic cells, the differentiation hierarchy of normal cells is in equilibrium, i.e. the abundances and proportions of different cell types do not change. Once the first leukemic stem cell arises, it produces a differentiation hierarchy of leukemic cells. The BCR-ABL oncogene is assumed to increase the rate at which progenitors and differentiated cells are being produced, 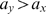 and 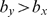. We assume that diagnosis occurs and treatment initiates once the leukemic cell burden reaches a threshold of 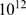 cells. Imatinib therapy counteracts the effects of BCR-ABL by reducing the differentiation rates to 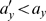 and 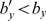, and possibly reducing the growth rate of cycling leukemic stem cells to 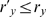. These effects result in distinct phases of exponential decline: (i) the first phase, with a slope of 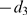, represents the depletion of terminally differentiated leukemic cells; since these cells have an average lifespan of about a day, their depletion cannot be observed in clinical data (IRIS trial) for reasons of low resolution [16]; (ii) the second phase, with a slope of 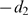, corresponds to the decline of differentiated leukemic cells; this slope can be observed in the IRIS data and has an average of 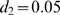 per day, suggesting that differentiated cells live approximately 20 days during therapy; (iii) the third phase, with a slope of 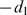, signifies the depletion of leukemic progenitors; this slope is about 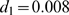, representing a lifespan of 125 days for progenitors during treatment; and (iv) the fourth phase reflects the effect of imatinib on cycling stem cells. If imatinib is unable to deplete cycling leukemic stem cells (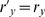), then the leukemic cell burden increases during the fourth phase, while it decreases if imatinib is capable of depleting cycling leukemic stem cells. The slope of the fourth phase of decline represents the rate of decline of leukemic stem cells and is calculated in the following.

**Table 1:** Basic model of differentiation hierarchy of normal and leukemic cells.

	Normal	Leukemic
Cycling stem cells	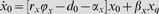	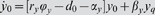
Quiescent stem cells	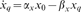	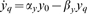
Progenitors	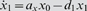	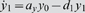
Differentiated cells	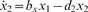	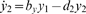
Terminally diff. cells	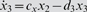	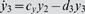

Denote by 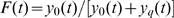 the ratio of cycling to total leukemic stem cells; the asymptotic value of this ratio during treatment is given by 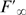. Note that if 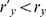, then 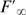 is not the same as the frequency of cycling stem cells at equilibrium (at which the system is initiated), since in that case imatinib therapy decreases the growth rate of cycling leukemic stem cells and disturbs the equilibrium of the system. To calculate the fourth slope, we isolate the two-dimensional system for 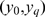 and consider the differential equation in terms of the ratio 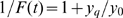. As treatment is administered and time increases, 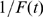 approaches 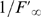, which is given by
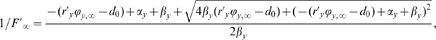
where 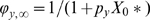 is the asymptotic value of 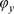 for large times. The equilibrium carrying capacity of the healthy cycling stem cells is given by 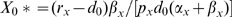. The slope of the fourth phase of decline of terminally differentiated cancer cells is then given by 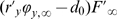. In Fig. 1B, we show the dynamics of cells observed in peripheral blood if cycling leukemic stem cells are slowly depleted by imatinib (lower line) and if cycling leukemic stem cells continue to increase during therapy (upper line).

## Results

### Parameter Sensitivity

**Figure 2:**
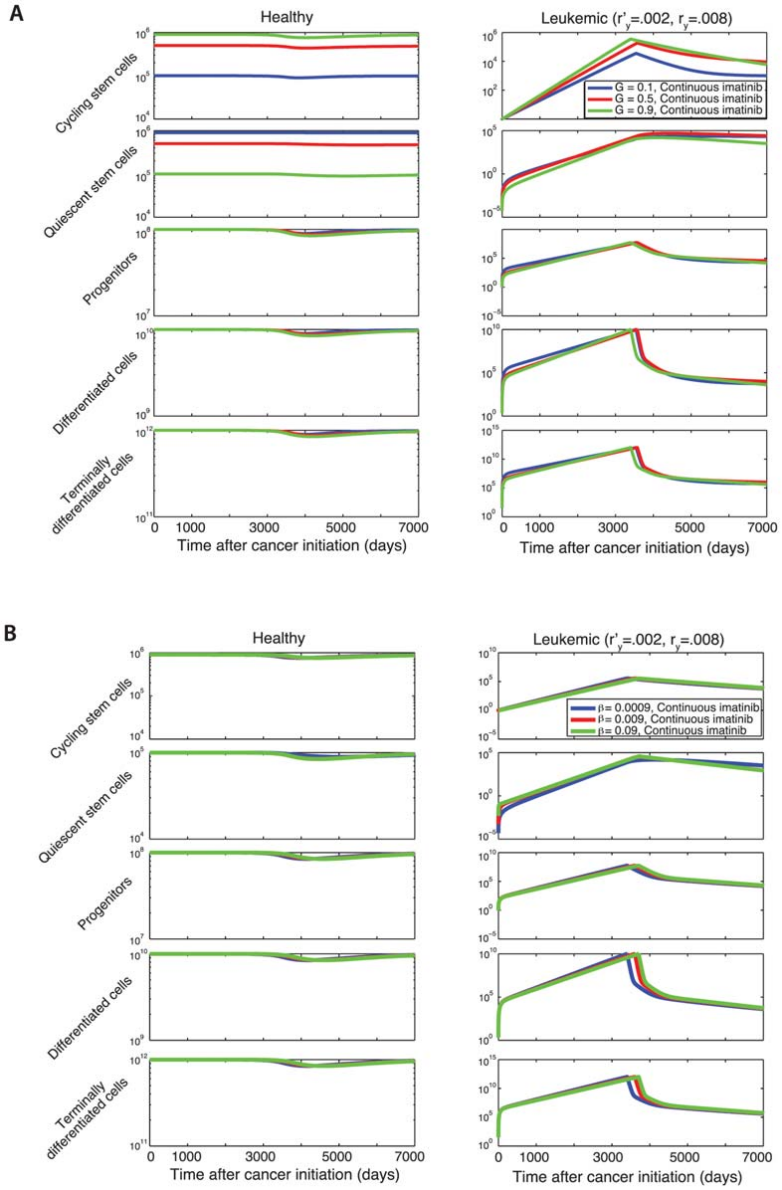
The effects of stem cell parameters on the model predictions. (A) We show the sensitivity of the model to the equilibrium frequency of cycling stem cells, 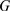. Initially, the healthy cells are in equilibrium and there is one leukemic stem cell. As the leukemic cell population increases, the healthy cell population begins to decrease due to competition. Treatment is initiated once the leukemic cell burden has reached a threshold of 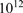 cells, at which point the leukemic cell burden begins to decline. Three scenarios are investigated: (i) the fraction of cycling stem cells is 10%, 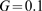, using values of 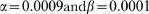 (blue lines), (ii) the fraction of cycling stem cells is 50%, 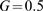, with 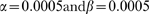 (red lines), and (iii) the fraction of cycling stem cells is 90%, 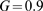, with 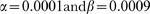 (green lines). Here 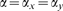 and 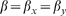, with all other parameters as in Fig. 1. (B) We show the sensitivity of the model to 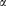 and 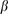 (i) 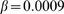 (blue lines), (ii) 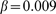 (red lines), and (iii) 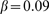 (green lines); in all cases 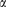 is chosen such that 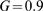. Note the relative insensitivity of number of terminally differentiated leukemic cells to 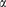 and 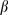.

Since the extent of quiescence among hematopoietic stem cells is the subject of controversy [21]–[24], we test the sensitivity of the model's predictions to changes in parameters. The parameter 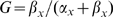 specifies the frequency of normal cycling stem cells if the system is in equilibrium; if 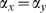 and 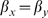, then 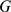 also denotes the equilibrium fraction of cycling leukemic stem cells. The effects of varying 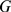 are shown in Fig. 2A where we plot the abundances of various cell types prior to and during imatinib therapy. Here we assume that diagnosis and treatment occur once the leukemic cell burden reaches a threshold of 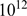 cells. We may also investigate the case in which the proportion of cycling stem cells to total stem cells is different for leukemic (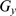) and normal (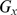) cells. In Figure S1 we investigate the dynamics of the system during imatinib therapy and during concurrent imatinib and G-CSF treatment when 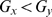. While changes in 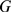 alter the abundance of cycling and quiescent stem cells, they do not modify the abundance of terminally differentiated leukemic cells by a significant amount. A choice of 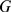 specifies the relationship between the rate at which cycling stem cells produce quiescent stem cells, 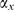 and 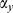 (for healthy and leukemic cells, respectively), and the rate at which quiescent stem cells produce cycling stem cells, 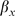 and 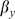. The effects of varying these turnover rates while keeping 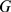 fixed prior to and during imatinib therapy are shown in Fig. 2B. The abundance of terminally differentiated cells is even less sensitive to such changes than to variation of 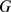. Therefore, despite insufficient knowledge about the extent and regulation of quiescence, a sensitivity analysis of the system determines that the model's predictions are very robust with regard to changes in parameters that determine the abundance and turnover of quiescent stem cells.

### Clinical Trial Results

**Figure 3:**
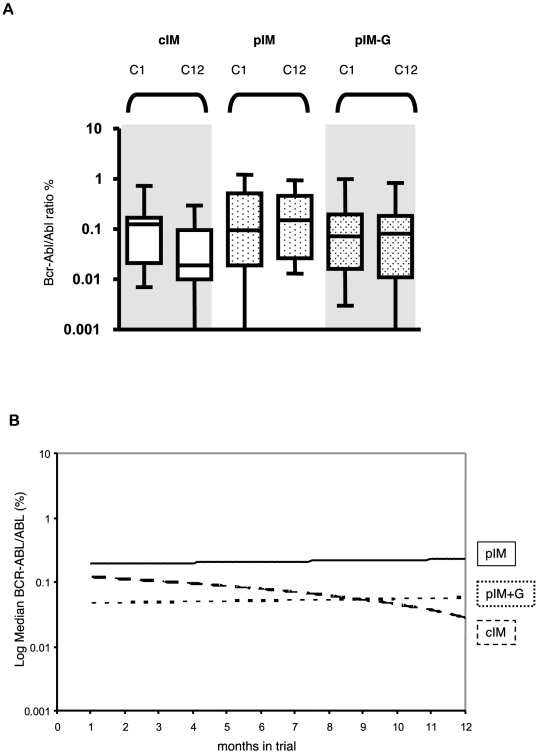
Results of a clinical trial administering continuous imatinib, pulsed imatinib, and pulsed imatinib with G-CSF. We show box and whisker plots of log *BCR-ABL/ABL* (%) levels (A) and plots of median log *BCR-ABL/ABL* (%) levels (B) for each study arm of the GIMI trial (see text for trial description). No significant differences were observed at cycles 1 (C1) or 12 (C12) as compared to baseline for either the pulsed imatinib arm or the pulsed imatinib with G-CSF when compared to the arm administering continuous imatinib as shown in (A) (p-values all >0.1). The trendlines in (B) demonstrate no significant differences between the arms when changes from baseline were compared (p-values all >0.1). ANCOVA analysis was used thereby adjusting results by their baseline values. Note that the average levels of patients in the pulsed imatinib arm and the pulsed imatinib with G-CSF arm differed at the outset of the trial but did not change during the study period. IM, imatinib mesylate; G, G-CSF.

In light of *in vitro* data supporting such a strategy [42], a randomized, phase II pilot clinical trial was commenced in 2004 to establish the safety of pulsed imatinib in combination with G-CSF. Imatinib interruption (for 1 week every 4 weeks) was deemed to be necessary based upon *in vitro* data suggesting an anti-proliferative effect of this strategy [31]. The trial was performed as a three arm, multi-center study which enrolled 15 patients in each arm: continuous imatinib (cIM) vs. pulsed IM (pIM) vs. pIM plus G-CSF (pIM-G) given three times weekly during the period of dose interruption. Results of the study have recently been published [32]. Monthly Q-PCR monitoring was performed over the 12 month study period and the results are summarized in Figs. 3A and 3B (unpublished observations). Although not powered to detect small differences between the treatment arms over time, no significant changes in BCR-ABL/ABL ratios (%) were noted over the study period. Furthermore, 6 patients in the experimental arms exhibited disease progression or loss of response, as compared to a single patient in the control arm (cIM). While this was not in itself statistically significant, the majority of these patients (5/7) had disease control re-established by reintroduction of daily TKI therapy suggesting that the experimental approach had contributed to the loss of response.

### Application of the Model to Clinical Trial

**Figure 4:**
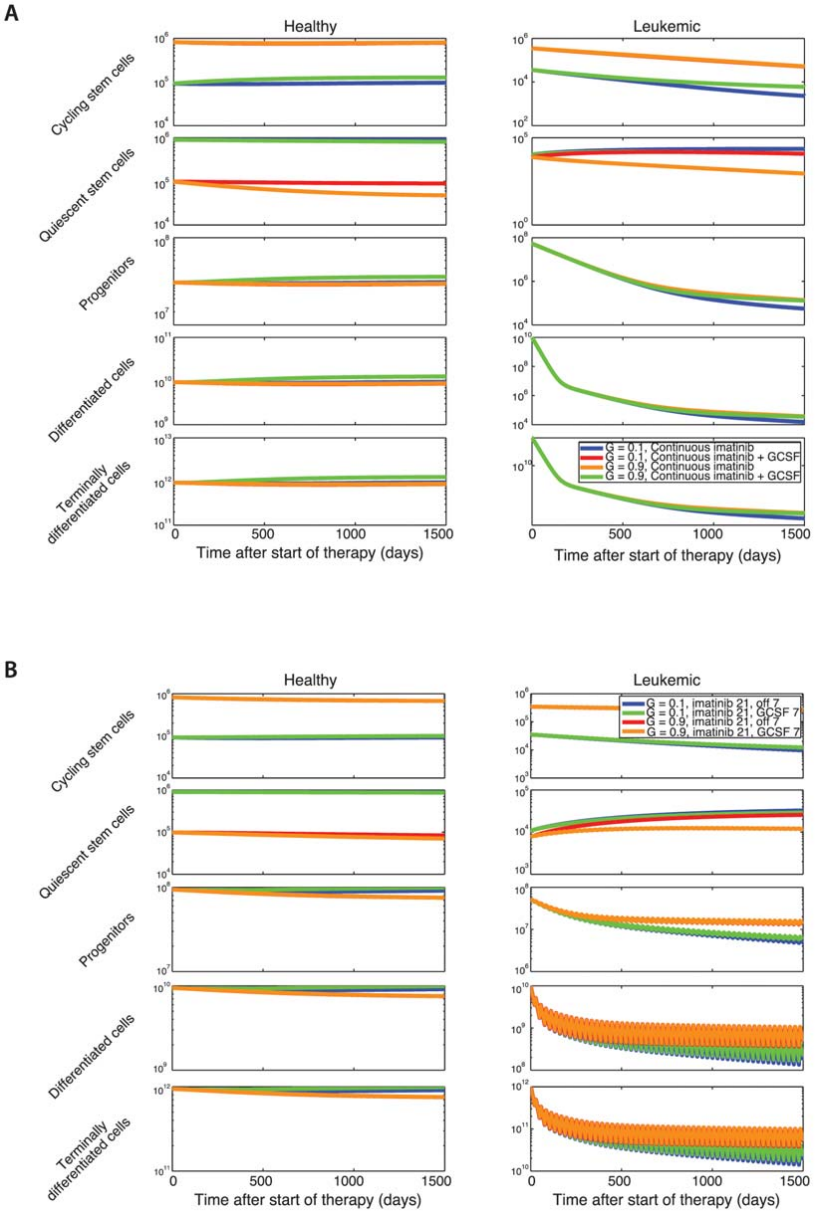
Treatment with imatinib and G-CSF. (A) The dynamics of the hematopoietic system during therapy with imatinib alone (blue and red lines) or imatinib in combination with G-CSF (green and orange lines) are shown. We investigate the system for two different equilibrium frequencies of cycling stem cells: *G* = 0.1 (blue and green lines), and *G* = 0.9 (red and orange lines). The addition of G-CSF has an effect on the abundance of terminally differentiated cells only if the fraction of cycling stem cells is small, *G* = 0.1. Parameter values during imatinib and G-CSF combination therapy are 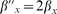 and 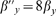; all other parameters are as in Fig. 2. (B) The dynamics of the hematopoietic system during therapy with pulsed imatinib (3 weeks on, one week off; blue and red lines) or imatinib alternating with G-CSF (3 weeks imatinib, one week G-CSF; green and orange lines) are shown. We investigate the system for two different equilibrium frequencies of cycling stem cells: *G* = 0.1 (blue and green lines), and *G* = 0.9 (red and orange lines). Again, the addition of G-CSF has an effect only if *G* = 0.1. Parameter values during G-CSF therapy are as above.

Let us now use our mathematical model to predict the outcome of the clinical trial described above [32]. First, we investigate the dynamics of the system during combination therapy with imatinib and G-CSF. G-CSF functions by mobilizing both healthy and leukemic quiescent stem cells [29]. In the context of our model, this effect corresponds to increasing 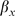 and, to an even larger extent, 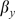. More specifically, we assume that G-CSF has a stronger effect on leukemic stem cells than on healthy stem cells [42]. The treatment response to imatinib alone and imatinib in combination with G-CSF is shown in Fig. 4A, where the abundances of each cell type are plotted as a function of time elapsed since the start of therapy. We assume that imatinib has no effect on the normal hematopoietic system (Fig. S2) and study two scenarios: (i) the majority of stem cells (both normal and leukemic) are initially quiescent [21],[22], 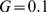, and (ii) the majority of stem cells are cycling [23],[24], 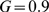. In the latter case, the addition of G-CSF does not change the dynamics of terminally differentiated cells during therapy at all. If most stem cells are quiescent, however, then the addition of G-CSF leads to an increase in cycling leukemic stem cells since there is an enhanced production of cycling stem cells from the quiescent pool; this effect translates into higher levels of terminally differentiated cells during therapy. Additionally, administration of G-CSF leads to an enhanced production of normal cycling stem cells from the quiescent pool, albeit to a lesser extent than in the leukemic system.

Next, we investigate the dynamics of the system during pulsed imatinib therapy with and without G-CSF. In Fig. 4B, we compare the effects of different treatment options on the abundance of terminally differentiated cells. We perform the comparison for the two cases in which the initial fraction of cycling stem cells is 10% and 90%, respectively (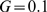 and 0.9). The outcome of such treatment choices is similar to the continuous treatment scenarios shown in Fig. 4A: if 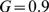, then the effects of adding G-CSF are negligible for the abundance of terminally differentiated leukemic cells; if 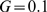, however, then the level of terminally differentiated leukemic cells is higher when G-CSF is administered than when imatinib is used alone.

**Figure 5:**
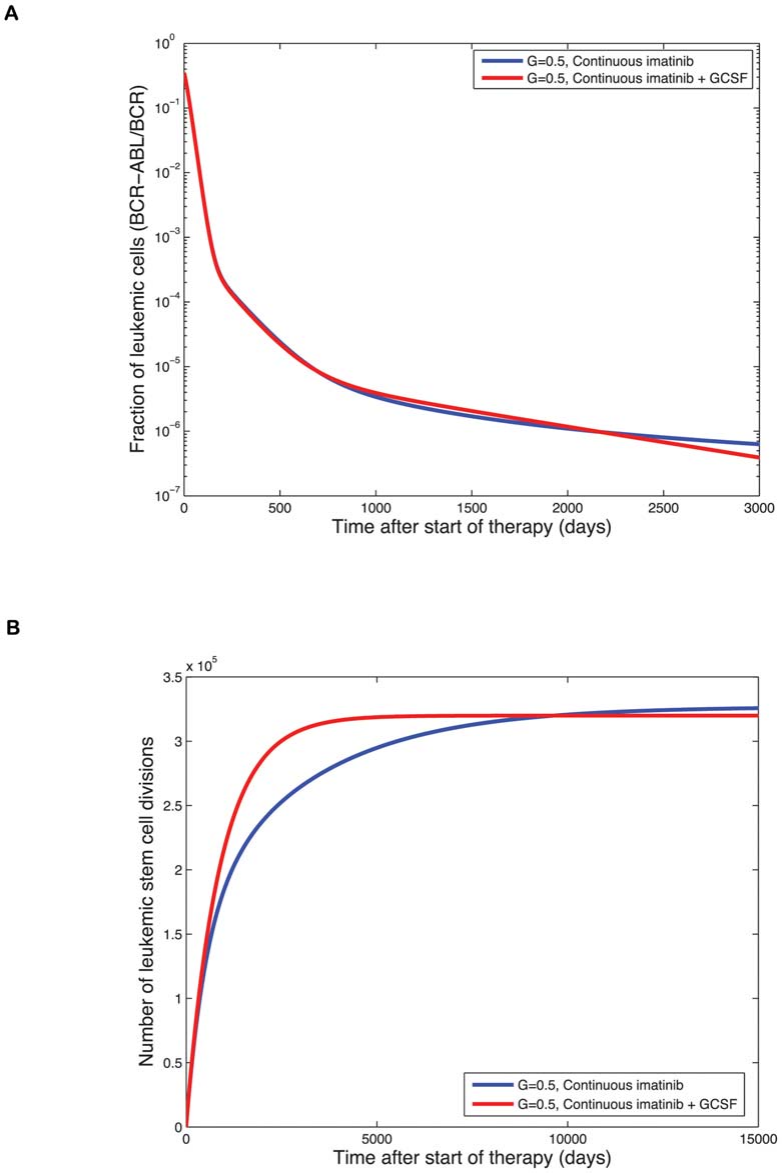
Long-term effects of therapy. (A) We show the long-term dynamics of leukemic cells in peripheral blood, BCR-ABL/BCR (%), during continuous imatinib therapy with (red line) and without (blue line) G-CSF. We plot 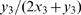 since normal cells have two alleles of BCR while leukemic cells have only one. Treatment starts on day zero and leads to a tri-phasic depletion of the leukemic cell burden if imatinib is capable of depleting leukemic stem cells. Although the addition of G-CSF initially increases the number of terminally differentiated leukemic cells, the long-term trend reveals that adding G-CSF does result in a faster rate of depletion of the leukemic cell burden and eventually leads to smaller numbers of terminally differentiated leukemic cells. The initial equilibrium frequency of cycling stem cells is 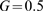 with 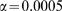 and 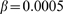 (all other parameters are as in Fig. 4). (B) We show the long-term simulation results for the number of leukemic stem cell divisions during continuous imatinib therapy with (red line) and without (blue line) G-CSF. This number is directly proportional to the risk of resistance to therapy. Until the crossover point of about 10,000 days, imatinib therapy leads to a lower number of stem cell divisions and therefore to a lower probability of resistance. After the crossover point, combination therapy with imatinib and G-CSF leads to a lower risk of resistance. All parameter values are as above.

So far we have found that depending on the abundance of quiescent stem cells, the addition of G-CSF to the imatinib treatment protocol either increases or does not affect the level of terminally differentiated leukemic cells in the first 1500 days of therapy. Note that these results are dependent upon our assumption that imatinib treatment leads to a decline of leukemic stem cells. We next investigate the long-term effects of continuous imatinib treatment with and without G-CSF (Fig. 5A). The addition of G-CSF is ultimately beneficial for the patient since it decreases the leukemic cell burden at a faster rate than imatinib therapy alone. However, for an intermediately long treatment horizon, the additional administration of G-CSF increases the leukemic cell burden since G-CSF enhances the production of cycling leukemic stem cells from their quiescent counterparts. This increased frequency of cycling stem cells allows for an enhanced effect of imatinib, leading to an exhaustion of both cycling and quiescent stem cells.

The evolution of point mutations in the BCR-ABL kinase domain conferring resistance represents a limitation for the usefulness of imatinib therapy [43]–[47]. We sought to investigate whether the addition of G-CSF to imatinib treatment modulates the risk of resistance in treated patients. Denote by 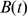 the number of cell divisions of leukemic stem cells until time *t*; this quantity can be calculated as 
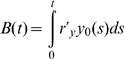
, which depends on the effects of imatinib (and G-CSF) therapy. The probability that a point mutation conferring resistance arises per stem cell division is denoted by 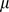. Here we assume that only leukemic stem cells can accumulate resistance mutations since they are the only cells that have self-renewal potential; if a resistance mutation arises in a progenitor or differentiated cell, then this resistant cell cannot produce a self-sufficient clone and will eventually die out. We also only consider resistance arising due to BCR-ABL kinase domain mutations and exclude other reasons for the emergence of resistance from our analysis. Then the probability that at least one resistant leukemic stem cell has arisen in a patient before time *t* is given by 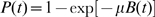. Thus a larger 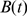 results in a higher risk of developing resistance by time 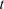. Note that this probability includes only newly emerging resistance and does not capture the risk of resistance due to mutations present prior to the initiation of therapy; since we are interested only in the effects of different treatment protocols on the risk of resistance and we assume complete resistance, we can neglect the latter type of mutations. In Fig. 5B we show the number of leukemic stem cell divisions, 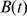, as a function of time for treatment with imatinib alone or in combination with G-CSF. There exists a crossover point in time, prior to which patients have a lower probability of developing resistance during imatinib therapy. Beyond the crossover point, combination therapy leads to a lower probability of resistance.

As a numerical example, consider the risk of resistance for patients 2500 days (∼6.8 yrs) after the initiation of therapy. If resistance can emerge due to any one out of 90 different point mutations in the BCR-ABL kinase domain and the baseline mutation rate is 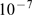 per base per cell division [48], then the probability that a resistance mutation arises per cell division is 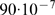. The model predicts that 89 out of every 100 patients would develop resistance if treated with imatinib alone, whereas 96 out of every 100 patients would develop resistance if treated with imatinib and G-CSF. If we assume a baseline point mutation rate of 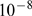 per cell division, then 20 out of 100 patients on imatinib and 27 out of 100 patients on imatinib and G-CSF would develop resistance during the first 2500 days of therapy. Our model predicts that patients on combination therapy who do not develop resistance will benefit from the later effects of G-CSF that reduce the levels of leukemic cells in the blood; however, they will remain at prolonged elevated risk of resistance in comparison to patients on imatinib therapy until the crossover point.

Similarly to the risk of resistance, we can also consider the risk of progression to accelerated phase and blast crisis during different treatment options. Using the model designed in [38], we predict that the rate of progression is lowest during treatment strategies that most effectively deplete progenitor cells if blast crisis stem cells arise from the progenitor pool [49]. Therefore, a treatment strategy that increases cycling leukemic stem cells and progenitors even transiently (such as G-CSF therapy) will increase the initial risk of progression to blast crisis. If G-CSF administration additionally protects progenitor cells from being inhibited by imatinib therapy [50], then the risk of progression is even more pronounced.

## Discussion

In this paper, we have presented a novel mathematical model of the hematopoietic system of CML patients during therapy. We have used this model to investigate the response to treatment with imatinib and/or the growth factor G-CSF. We have studied four different treatment strategies: (i) continuous administration of imatinib alone; (ii) continuous administration of combination therapy with imatinib and G-CSF; (iii) pulsed imatinib followed by a treatment break; and (iv) pulsed imatinib followed by G-CSF therapy. The capability of G-CSF therapy to modulate the leukemic cell burden depends on the extent of quiescence among leukemic stem cells: if the majority of leukemic stem cells are quiescent, then the addition of G-CSF to imatinib therapy temporarily increases the leukemic cell burden in peripheral blood. Eventually this treatment strategy leads to a more rapid decline of leukemic cells. However, this effect is only observed if imatinib is capable of depleting cycling leukemic stem cells. If cycling stem cells are insensitive to imatinib *in vivo*, then the addition of G-CSF will only increase the leukemic cell burden. Furthermore, we have not considered a protective effect of G-CSF on leukemic stem cells [50] since cytokines seem to protect only bulk CD34+ cells from tyrosine kinase inhibition and about 10% of primitive CML stem cells survive a 12 day exposure to dasatinib in the absence of any added cytokines [51]. However, the inclusion of such an effect would make our conclusion even stronger that the addition of G-CSF to tyrosine kinase inhibitor (TKI) therapy may not be beneficial in the clinic.

The effects of therapy with continuous and pulsed imatinib as well as pulsed imatinib with G-CSF have been investigated in a pilot study in 2009 [32]. Forty-five patients were randomized between three treatment arms: continuous imatinib, pulsed imatinib, and pulsed imatinib with G-CSF. Since the patients recruited to participate in this pilot study were not newly diagnosed but already pre-treated with imatinib, their cell counts had already reached the fourth phase in the dynamics (see Fig. 1B). Our mathematical model predicts differences in the leukemic cell burden between patients on imatinib therapy with and without G-CSF only if the majority of leukemic stem cells are quiescent (Fig. 4). If most leukemic stem cells are cycling, then the addition of G-CSF to imatinib therapy is expected to have no appreciable effect on the BCR-ABL RQ-PCR levels. This situation was observed in the pilot study [32], suggesting that the majority of leukemic stem cells are cycling. This conclusion, however, can only be drawn if imatinib is capable of inhibiting cycling leukemic stem cells.

Lastly, we have determined the risk of resistance arising during imatinib therapy with and without G-CSF. While treatment with imatinib alone eventually leads to a higher risk of resistance, combination therapy with imatinib and G-CSF initially confers a larger probability of acquired resistance. A similar conclusion can be drawn regarding the risk of progression to accelerated phase and blast crisis.

Since the trial was designed to investigate the safety of this treatment protocol and was not powered for efficacy, the conclusions about response and disease progression should not be over-interpreted. Although the PCR values over time were not significantly different between the three treatment arms, the continuous imatinib arm tended downwards during the duration of the trial, while the other two arms did not show any increases or decreases in leukemic cell burden (Fig. 3B). This observation is expected from the model since discontinuous administration of imatinib should show a smaller effect on the disease burden than continuous administration if imatinib therapy is capable of inhibiting leukemic stem cells. Also, 6 of 7 cases showing disease progression were in the pulsed treatment arms; this effect is also in concordance with our model's prediction since the risk of resistance is directly correlated with the number of leukemic stem cells which, if sensitive to imatinib, are less abundant in patients receiving continuous therapy than in those receiving pulsed doses.

Our findings regarding the risk of resistance and progression together with the absence of a clinical response with the addition of G-CSF suggest that this treatment option may not be beneficial for CML patients in the clinic.

## Supporting Information

Figure S1 Equilibrium frequency of normal vs. leukemic cycling stem cells. We compare dynamics of the model in two cases: (i) the frequency of cycling leukemic stem cells, Gy = 0.9, is greater than the frequency of cycling healthy stem cells, Gx = 0.5, and (ii) the cycling frequencies of normal and leukemic stem cells are the same (Gx = Gy = 0.9). All other parameter values are as above. In (A) we show the model predictions prior to and during continuous imatinib therapy, and in (B) we show the analogous results for continuous imatinib plus G-CSF therapy. Note that although the change in Gx changes the abundances of quiescent and cycling normal stem cells, we do not see much change in the total leukemic cell burden in either case.
(1.01 MB TIF)

Figure S2 Effect of imatinib on normal hematopoietic stem cells. We investigate the dynamics of the model if imatinib has the additional effect of slightly lowering the growth rate of normal hematopoietic stem cells (rx' = 0.0045<rx = 0.005) and compare this situation to the case when imatinib has no effect on these cells (rx' = rx = 0.005). We observe that this modification does not significantly alter the leukemic cell burden.
(0.86 MB TIF)
