# Supplementary figures and images for: Eradication of Chronic Myeloid Leukemia Stem Cells: A Novel Mathematical Model Predicts No Therapeutic Benefit of Adding G-CSF to Imatinib
Source: PLoS Comput Biol. 2009 Sep 11;5(9):e1000503. doi: 10.1371/journal.pcbi.1000503 (PMC2730033; doi:10.1371/journal.pcbi.1000503)

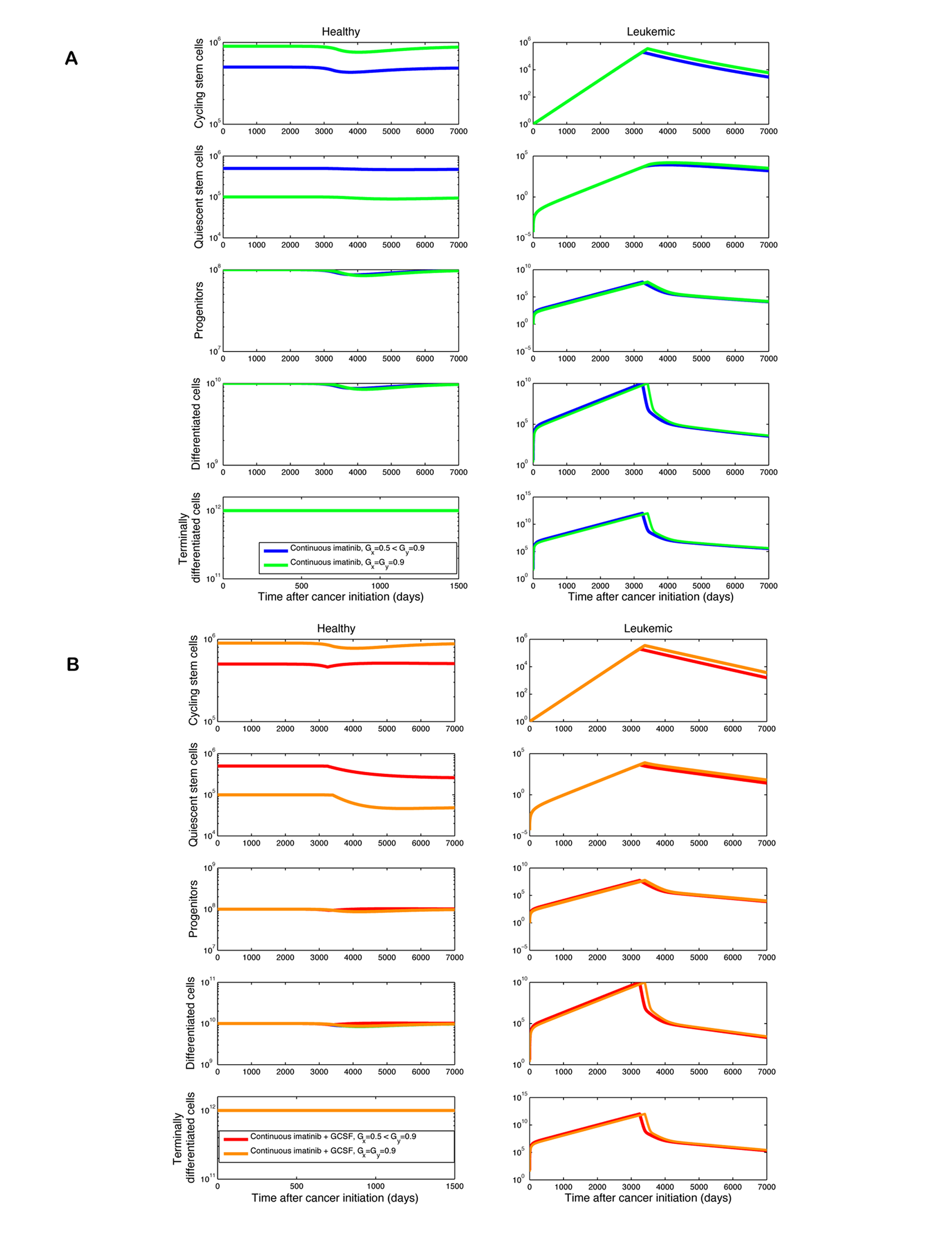

Supplement: Figure S1 — Equilibrium frequency of normal vs. leukemic cycling stem cells. We compare dynamics of the model in two cases: (i) the frequency of cycling leukemic stem cells, Gy = 0.9, is greater than the frequency of cycling healthy stem cells, Gx = 0.5, and (ii) the cycling frequencies of normal and leukemic stem cells are the same (Gx = Gy = 0.9). All other parameter values are as above. In (A) we show the model predictions prior to and during continuous imatinib therapy, and in (B) we show the analogous results for continuous imatinib plus G-CSF therapy. Note that although the change in Gx changes the abundances of quiescent and cycling normal stem cells, we do not see much change in the total leukemic cell burden in either case. (1.01 MB TIF) [file pcbi.1000503.s001.tif]

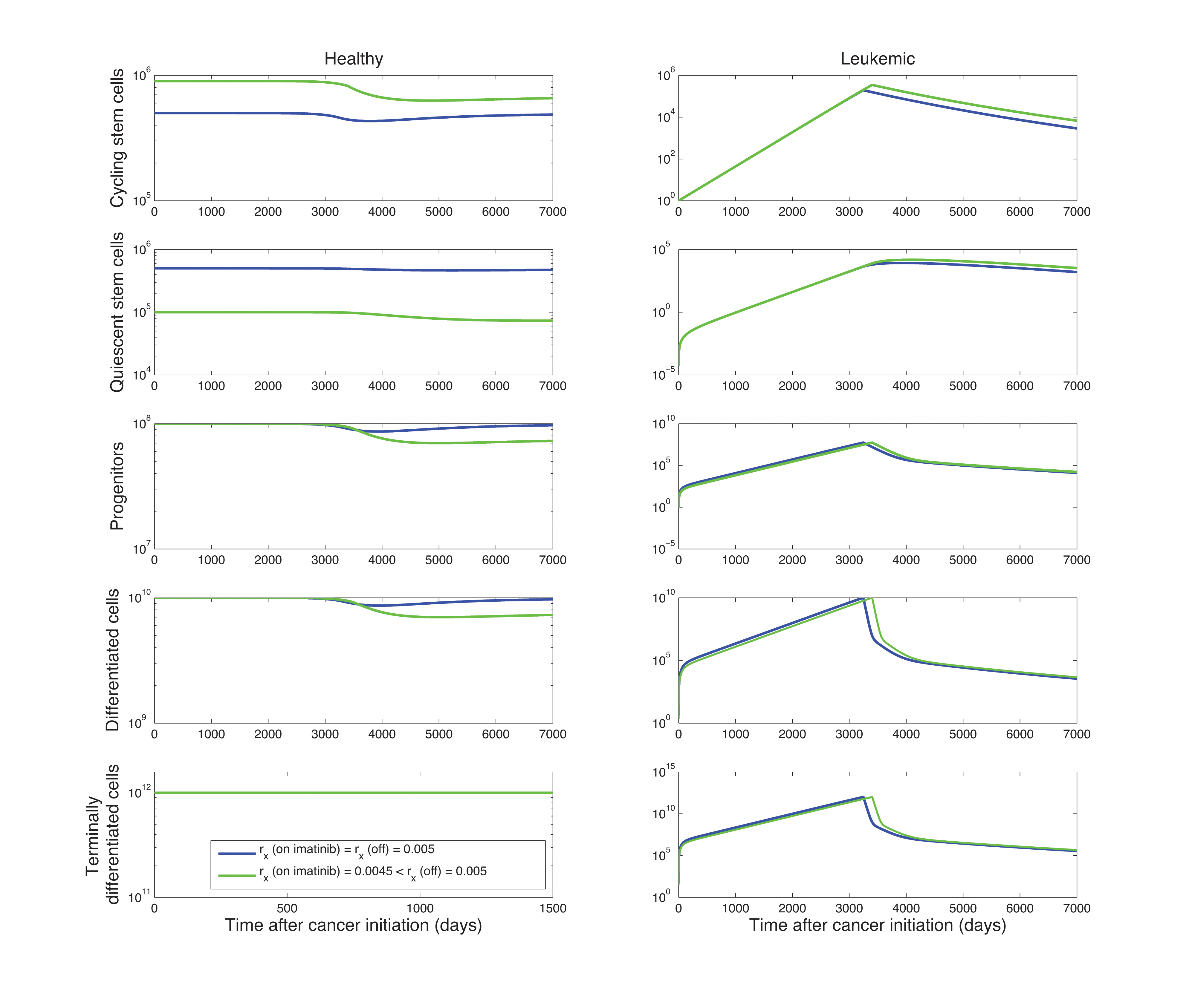

Supplement: Figure S2 — Effect of imatinib on normal hematopoietic stem cells. We investigate the dynamics of the model if imatinib has the additional effect of slightly lowering the growth rate of normal hematopoietic stem cells (rx' = 0.0045<rx = 0.005) and compare this situation to the case when imatinib has no effect on these cells (rx' = rx = 0.005). We observe that this modification does not significantly alter the leukemic cell burden. (0.86 MB TIF) [file pcbi.1000503.s002.tif]
